# Ambient air pollution undermines chemosensory sensitivity – a global perspective

**DOI:** 10.1038/s41598-024-75067-z

**Published:** 2024-12-16

**Authors:** Anna Oleszkiewicz, Andrea Pozzer, Jonathan Williams, Thomas Hummel

**Affiliations:** 1 Smell and Taste Clinic, TU Dresden, Dresden, Germany; 2 Institute of Psychology, University of Wroclaw, ul. Dawida 1, Wroclaw, 50-527 Poland; 3 Max Planck Institute for Chemistry, Mainz, Germany; 4 Climate and Atmosphere Research Center, The Cyprus Institute, Nicosia, Cyprus

**Keywords:** Olfaction, Pollution, Atmospheric chemistry, Olfactory sensitivity, Multicentre study, Urban ecology, Human behaviour, Neurophysiology

## Abstract

This study offers insights into the complex relationship between chemical species constituting air pollution and chemosensory function. We examined the relationship between chemical species known to contribute to air pollution and assault human health and chemosensory sensitivity. Chemosensory sensitivity data was retrieved from a large-scale study involving 711 urban-dwelling participants inhabiting 10 different regions of the globe. Their olfactory threshold towards phenyl ethyl alcohol (PEA) and olfactory/trigeminal threshold towards Eucalyptol was measured in a multicentre study. We matched the individual chemosensory data with the levels of PM2.5, PM10, O3, NO2, SO2, CO at the location of testing sites, on the exact date of the test, using EMAC (ECHAM5/MESSy for Atmospheric Chemistry) model. Our findings indicate that air pollution negatively affects olfactory function and has cumulative negative effects with aging. The reported patterns are seasonal and increase during Autumn and Winter, and interact with medical conditions related to poorer olfactory function. We extend the current knowledge by demonstrating that olfactory/trigeminal perception is also disrupted by toxic air, albeit in a slightly different manner. The analyzed models promote a more complex perspective on the relationship between air composition and chemosensory sensitivity, but delineate problems related to the interdependence of the levels of chemical species constituting air pollution and using them together to predict chemosensory sensitivity. Conclusions point to the need to investigate the problem of air pollution and chemosensory health from a global perspective, as air quality partly accounts for the differences in chemosensory perception in different regions of the world.

**Supplementary Information:**

The online version contains supplementary material available at 10.1038/s41598-024-75067-z.

## Introduction

Ambient air pollution becomes an increasing threat to human health. According to the World Health Organization over 99% of the global population is exposed to air quality not meeting the recommended standards^1^. Exposure to outdoor air pollution has been linked to the risk of cardiovascular disease^2^, stroke^3,4^, chronic obstructive pulmonary disease, lung cancer^5–8^, oxidative stress^9^ as well as an increase in the risk for acute respiratory infections^10,11^.

Increasing awareness of air pollution has also resulted in more attention being paid to the influence of ambient air pollution on the olfactory system, partly because the nasal cavity is where airborne pollutants make the first contact with human tissues. Olfactory receptor neurons (ORNs), located in the olfactory mucosa on the roof of the nasal cavity, are among the most exposed neurons in the human body, separated from the environment by only a thin layer of mucus^12^. Thereby ORNs have been considered an ‘open window’ for airborne pollutants to directly translocate along the olfactory nerves, bypass the blood–brain barrier, and cause neurogenic inflammations^13–15^. Fine particulate matter (i.e. particle with an aerodynamical diameter below 2.5 micrometer, PM2.5) causes sinonasal and mucosal inflammation, resulting in dysplastic changes in the olfactory epithelium^16^. Post-mortem evidence from people exposed to high levels of airborne pollutants shows the accumulation of β-amyloid, cyclooxygenase-2, particulate matter (PM), and metals in the olfactory bulb and frontal lobe, suggesting further insult to the central nervous system^17–20^. Less is known about the behavioural consequences of airborne pollution exposure.

Olfactory deficits may occur three times more often in urban populations exposed to ambient air pollution, as compared to populations breathing relatively clean air^17^. Exposure to extreme ambient air pollution is associated with decreased olfactory sensitivity, the ability to differentiate odors (Hudson et al.^21^), and odor identification performance^22^. Conversely, indigenous tribes inhabiting the least polluted areas have been found to have a better olfactory function in comparison to industrialized populations, likely due to the lesser exposure to airborne pollutants^23–27^. The effect of airborne pollution on olfactory function is cumulative, meaning that its impact increases over the lifespan^28^. Indeed, cross-sectional^29^ and longitudinal (Ekström et al.^30^) studies demonstrated a progressive adverse effect of air pollution exposure in older populations, even when the pollution levels were only slightly exceeding WHO standards^31^. Olfactory loss is an important predictor of 5-year mortality risk in older individuals^32–38^ and there is speculation that air pollution may contribute to this effect. However, direct evidence supporting this connection is still lacking. Indirectly, the recent pandemic demonstrated the relationship between exposure to airborne pollutants (such as SO2 and CO) and the severity of SARS-CoV-2-induced chemosensory loss and mortality^39–42^. The presumed mechanism explaining elevated susceptibility to respiratory viral infections refers to the altered viral life cycle and weakened immunological response. Exposure to air pollutants may promote viral entry by inhibiting the antiviral activity of the surfactant proteins D and antimicrobial peptides and increasing levels of ACE2 receptors. Exposure to toxic air pollutants may reduce ATP regeneration, stimulate apoptosis, and induce autophagy. Coronaviruses may then use autophagy to generate double-membranous vesicles and consequently benefit replication^43^. The proposed mechanisms require more empirical proof, but if confirmed, may explain enhanced virus-induced tissue inflammation and damage, and weakened immunological response translating to significant behavioural consequences of infection as an effect of air pollution exposure. Additionally, the cumulative effect of air pollution on COVID-19-related mortality may also be mediated by pre-existing comorbidities such as cardiopulmonary diseases^44,45^. The cumulative effect of PM2.5 exposure has been shown to have a negative effect on olfactory identification abilities followed by an increased risk of dementia, especially in APOE ɛ4 allele carriers and individuals with relatively low olfactory performance at the baseline^31^.

One way to deepen our understanding of the role of environmental factors affecting the human sense of smell is to study the relationships between exposure to air pollution and chemosensory functions. To date, conclusions are limited to certain locations and prevent broader reasoning about the impact of air pollutant species on the olfactory system. Comparative studies have selected populations based on their assumed exposure to air pollution and failed to precisely quantify the relationship between air pollution indices with olfactory functions. Instead, it led to the general conclusions on the olfactory performance as a function of high vs. low air pollution^17,21,23,27,46^. Moreover, air pollution is a complex mixture of a particular matter, chemicals and pathogens and may differ across geographical regions of the globe. It is a challenge to identify environmental factors that are particularly harmful to the sense of smell; nevertheless, the first steps have already been taken in this field. In general, researchers have focused on the chemical species that are the main constituents of air pollution with a proven adverse impact on human health, such as PM10, PM2.5, NO2, and O3. Beyond that, SO2 and CO have additionally been linked to COVID-19 incidence. Selected chemical species have been investigated in the context of human olfactory function with a primary focus on PM2.5, PM10 (a particle with an aerodynamical diameter below 10 micrometers), and nitrogen oxides (NOx) levels^30,47,48^. Studies on pollution-related olfactory dysfunction rarely include a variety of chemical species present in ambient air that are known to harm human respiratory health, but rather focus on particular chemical species. The current discussion is missing a more complex perspective on the impact of air quality on olfactory function, with a particular focus on olfactory sensitivity, being the primary function exposed to the toxic agents in the air. To address this problem, we matched global-scale olfactory sensitivity data^49^ with air pollution indices extracted from a global atmospheric chemical model to thoroughly catalogue the impact of air pollution on olfactory sensitivity. The current study focuses on chemosensory thresholds for olfactory and mixed olfactory/trigeminal stimuli because thresholds represent the function of the peripheral sensory system at a mucosal level to a higher degree than other measures of chemosensory function, like odor identification^50^. This is done because the mucosa is the location where toxic air makes the first contact with the human body. The intranasal trigeminal system mediates chemically induced sensations such as stinging, burning, or cooling, while the olfactory system decodes smell quality^51^.

It is expected to observe an overall negative relationship between the levels of PM2.5, PM10, O3, NO2, SO2, CO, and chemosensory sensitivity. As these six chemical species have never been considered jointly in the context of olfactory performance, the study aims to quantify the impact of each of these chemical species known to contribute to air toxicity and likely to be harmful to the sense of smell. In this work, we refer to both olfactory/trigeminal and olfactory systems. By looking into trigeminal/olfactory and olfactory systems jointly we aimed to explore the assumption that these two systems, although closely related^52–56^, may be differently impacted by air pollution. No directional hypotheses are proposed in this regard.

## Results

### Model fit

As evidenced in Table 1, adding each group of predictors (Air pollution indices, Self-rated olfactory function, Season and Location) to the baseline models systematically improved model fitness. Given the significantly lowest AIC values for models including Air pollution indices, Self-rated olfactory function, Season and Location, these variants were chosen for further analysis concerning olfactory/trigeminal and olfactory thresholds.

**Table 1:** Model fit comparisons between the baseline model and the seven alternative models including three groups of predictors: air pollution indices, self-rated olfactory function (including interaction terms with gender, Age, and medical conditions), season and location.

Air pollution	Self-assessed olfactory function	Season and location	MAD	Person’s *r*	AIC	∆AIC	*p*	∆df
Olfactory/trigeminal sensitivity (Eucalyptol)								
N	N	N	1.54	0.09	8819.47			
N	Y	N	1.54	0.13	8713.63	− 105.84	< 0.001	4
N	N	Y	1.28	0.49	7237.37	− 1476.26	< 0.001	10
N	Y	Y	1.28	0.49	7179.46	− 57.92	< 0.001	4
Y	N	N	1.49	0.23	8456.91	1277.45	< 0.001	− 10
Y	Y	N	1.49	0.25	8345.09	− 111.82	< 0.001	4
Y	N	Y	1.26	0.51	7092.13	− 1252.96	< 0.001	9
**Y**	**Y**	**Y**	**1.26**	**0.52**	**7012.32**	**− 79.81**	**< 0.001**	3
Olfactory sensitivity (PEA)								
N	N	N	1.76	0.10	10438.22			
N	Y	N	1.70	0.22	10068.24	− 369.99	< 0.001	4
N	N	Y	1.34	0.60	7709.93	− 2358.31	< 0.001	9
N	Y	Y	1.33	0.61	7587.95	− 121.97	< 0.001	4
Y	N	N	1.61	0.38	9346.85	1758.90	< 0.001	-10
Y	Y	N	1.58	0.42	9084.84	− 262.01	< 0.001	4
Y	N	Y	1.33	0.60	7640.48	− 1444.36	< 0.001	8
**Y**	**Y**	**Y**	**1.32**	**0.61**	**7497.70**	**− 142.79**	**< 0.001**	4

The best-fit model (the lowest AIC) was selected for further analyses to assess the predictive value of Air Pollution, Self-assessed olfactory function, season and location for olfactory/trigeminal and olfactory sensitivity.

Y – Yes, group of predictors included in the model; N – No, group of predictors not included in the model; MAD – Mean Absolute Deviation; AIC – Akaike’s Information Criterion; ∆AIC – because we have compared models fitness in a systematic manner, the change in AIC represents the difference between the current model and the previous model (listed one row above in the table); ∆df – the difference in the number of predictors used in the model as compared to the previous model. ∆df differs between model steps for PEA and Eucalyptol as irrelevant predictors were automatically removed from the model.

### Predictors of the olfactory/trigeminal sensitivity (Eucalyptol)

#### Individual factors

The presence of smell-related medical conditions (β = − 0.67, *p* = .02) was negatively related to the sensitivity toward Eucalyptol. Age had non-significant adverse effects on the sensitivity to Eucalyptol for healthy women (β = − 0.004, *p* > .05) while sensitivity to Eucalyptol increased with age in healthy men (β = 0.005, *p* < .001). When accounting for smell-related medical conditions, both sexes were equally, negatively affected by olfactory aging (women: β = − 0.022, *p* < .001; men: β = − 0.019, *p* < .001). In other words, women with olfaction-related medical conditions would lose 0.022 points in the olfactory/trigeminal threshold test every next year of their life while for healthy women this loss would be only 0.002 points. Healthy men would gain additional 0.005 points in the olfactory/trigeminal test every next year of their life, while in the presence of medical conditions related to olfaction, men would lose 0.019 points in 8-point scale olfactory threshold test each year. Ratings of Eucalyptol Pleasantness (β = 0.05, *p* < .001) and Familiarity (β = 0.08, *p* < .001) were positively related to the sensitivity toward Eucalyptol. These ratings turned out to selectively moderate the effects of individual factors (Age, Gender, Medical conditions) on olfactory/trigeminal sensitivity, but the interpretation of these effects exceeds the scope of the current manuscript. All regression coefficients are summarized in Table S2A for transparency.

#### Self-rated olfactory function

People who rated their sense of smell better (β = 0.11, *p* < .001) were able to detect lower concentrations of Eucalyptol. The relationship between sensitivity to Eucalyptol and self-assessed olfactory function was weaker in healthy women (β = 0.11, *p* < .001) than men (β = 0.21, *p* < .001).

#### Season and location

People tested in Autumn (β = − 0.28, *p* < .001) and Winter (β = − 0.13, *p* = .009) performed worse in the olfactory/trigeminal sensitivity task than those who attended the study in Spring. Results obtained in the Summer were similar to those from the Spring (β = 0.004, *p* = .94). In reference to Poland, we found a significantly lower likelihood of higher olfactory sensitivity in Australia (β = − 0.38, *p* < .001) and a significantly higher likelihood for better scores in China (β = 0.29, *p* = .015) India (β = 0.78, *p* < .001), Iran (β = 0.61, *p* < .001), Japan (β = 2.05, *p* < .001), Mexico (β = 0.32, *p* < .001), Turkey (β = 0.74, *p* < .001) and the USA (β = 0.48, *p* < .001). For details regarding between-country variability in olfactory/trigeminal sensitivity see:^49^.

#### Air pollution

**Fig. 1:**
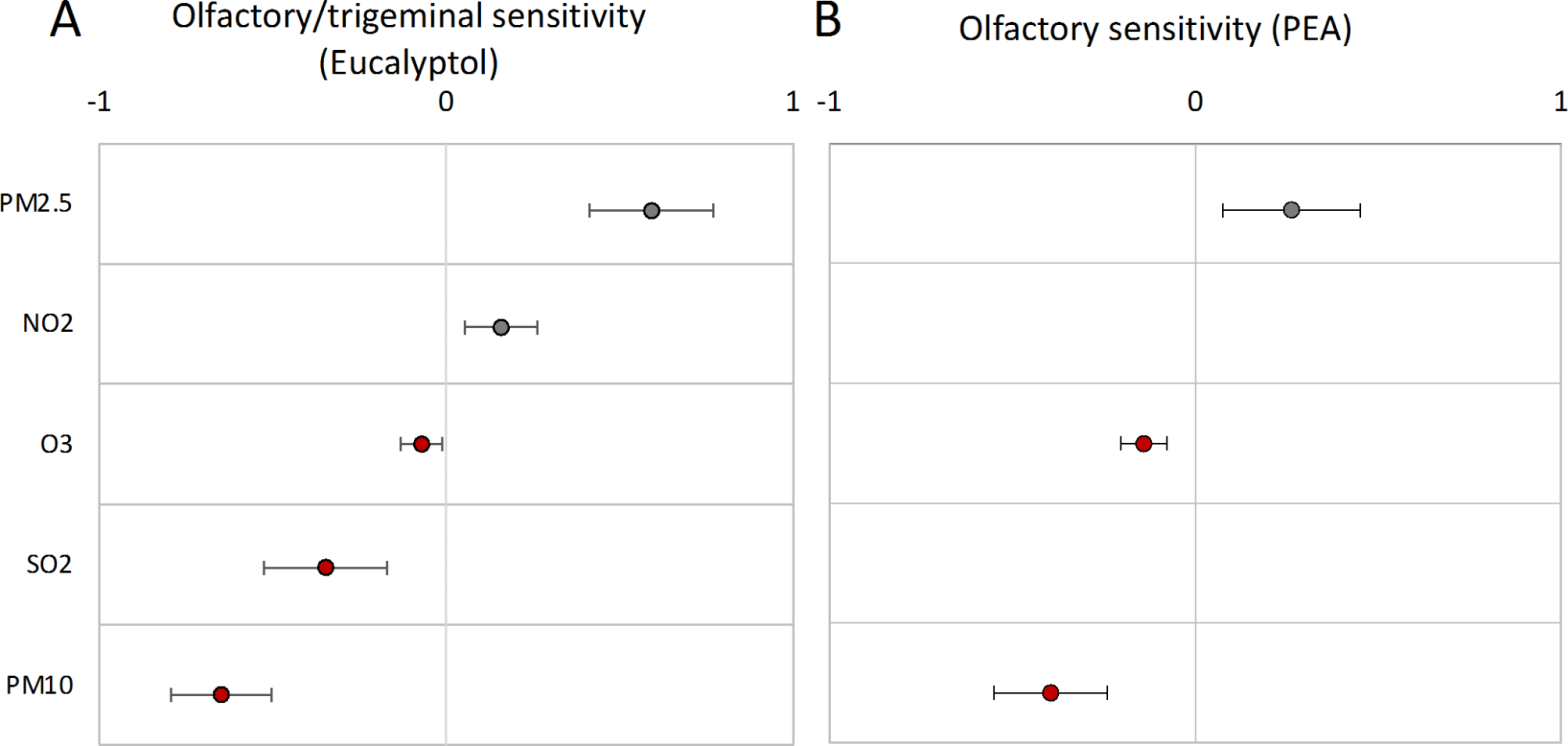
Effect sizes for the air pollution predictors of olfactory/trigeminal and olfactory sensitivity. Dots represent effect sizes (β) and whiskers denote ± 95% confidence intervals. Dots located between values − 1 and 0 (x-axis; red colour) are negatively related to olfactory/trigeminal or olfactory sensitivity; dots related between the values 0 and 1 (red colour) are positively related to olfactory/trigeminal or olfactory sensitivity.

All six species included in the model were relevant for olfactory/trigeminal sensitivity predictions, five being statistically significant with an exception for CO (β = 0.11, *p* = .10). Among these, PM10 (β = − 0.65, *p* < .001), SO2 (β = − 0.35, *p* < .001), O3 (β = − 0.07, *p* = .02) were negatively related to sensitivity to Eucalyptol, while PM2.5 (β = 0.60, *p* < .001) and NO2 (β = 0.16, *p* = .003) were positively associated with higher sensitivity to Eucalyptol. For graphical summary see Fig. 1A.

### Predictors of the olfactory sensitivity (PEA)

#### Individual factors

Age was negatively related with olfactory sensitivity (β = − 0.009, *p* < .001). The presence of medical conditions related to olfaction was associated with lower olfactory sensitivity (β = − 1.27, *p* < .001). Among healthy participants, only in women we observed olfactory aging (β = − 0.009, *p* < .001). In men, olfactory threshold scores were slightly increasing with age (β = 0.0014, *p* < .001). The presence of medical conditions affecting the sense of smell did not accelerate aging effects in women (β = − 0.008, *p* > .05), but men who reported olfaction-related medical conditions experienced accelerated olfactory aging effects (β = − 0.024, *p* < .001) in comparison to healthy men. Among psychophysical ratings of PEA, only pleasantness was negatively related to the sensitivity towards PEA (β = − 0.04, *p* = .02) indicating that lower sensitivity to PEA was associated with perceiving this odor as more pleasant. See Table S2B for all regression coefficients and their significance.

#### Self-rated olfactory function

Higher self-ratings of olfactory function were related to the greater sensitivity towards PEA (β = 0.20, *p* < .001). Self-ratings of olfactory function were not involved in any interaction effects predicting olfactory sensitivity.

#### Season and location

People tested in the Winter (β = − 0.30, *p* < .001) performed worse in the olfactory sensitivity task than those who attended the study in Spring. Consistently with results for olfactory/trigeminal sensitivity, compared to Poland, we found a significantly lower likelihood of higher olfactory sensitivity in Australia (β = − 0.69, *p* < .001) and a significantly higher likelihood for better scores in China (β = 1.36, *p* < .001), India (β = 1.47, *p* < .001), Japan (β = 1.97, *p* < .001), Mexico (β = 1.31, *p* < .001), Turkey (β = 1.10, *p* < .001) and the USA (β = 1.18, *p* < .001).

#### Air pollution

Among the six tested species, we noted significantly harmful effects of PM10 (β = − 0.40, *p* < .001), O3 (β = − 0.14, *p* < .001) and a positive relationship between the levels of PM2.5 and olfactory sensitivity (β = 0.26, *p* = .006). SO2, NO2 and CO were statistically irrelevant for predicting olfactory sensitivity. See Fig. 1B and Table S2B.

## Discussion

The current study corroborates former conclusions that human olfactory function is negatively impacted by some of the chemical species contributing to air pollution. Beyond that, it demonstrates that olfactory/trigeminal perception is disrupted by toxic air. The current study offers a unique comparison between olfactory and olfactory/trigeminal sensitivity in the context of air pollution exposure and concludes that these two systems are impacted by the chemical species constituting air pollution in a similar, yet not identical manner. In particular, our study demonstrates that NO2 and SO2 are relevant for predicting olfactory/trigeminal sensitivity, while they are unrelated to the olfactory sensitivity. Hence, the perception of odors as cooling, stinging, or burning can be modulated by more chemical species present in the air of poor quality. This may be particularly problematic for people with olfactory loss, whose chemosensory perception is based more on trigeminal function in comparison to individuals with normosmia, allowing for some compensation for the loss of olfactory sensitivity^57^. Inflammatory responses are likely to be the mechanism through which the trigeminal nerve is impacted by airborne toxic agents. Analogously in the clinical context, nasal allergies or chronic rhinosinusitis are associated with changes of the trigeminal system in terms of increased or decreased sensitivity, respectively^58^.

As expected, we observed that within the complex mixture constituting air pollution some chemical species were more harmful than others. We saw convergence between olfactory/trigeminal and olfactory sensitivity in terms of the negative impact of O3 and PM10 with the latter bearing the leading negative role. PM10 are relatively big particles, typically dust or sea salt, that are likely to dissolve in the mucus, change its characteristic, and result in impeded chemosensitivity. The chemical and physical mechanisms underlying the negative relationship between PM10 levels and chemosensitivity are yet to be unraveled. O3 is likely to have a damaging impact on olfactory nerves. Moreover, it is used as a primary predictor in calculating mortality risk due to pollution. SO2 was negatively related to olfactory/trigeminal sensitivity, but not olfactory sensitivity. This gas is soluble and may acidify the liquid-covered airways of the upper respiratory tract (i.e. it may deposit to the wet surfaces and makes small amounts of H2SO4 which may well cause irritation). This putative mechanism requires more careful monitoring of SO2 distribution in the upper respiratory tract to fully address the divergent effects on olfactory and olfactory/trigeminal sensitivity.

Perhaps the most surprising result from the current study is the positive relationship between the sensitivity to Eucalyptol and PEA, and levels of PM2.5 and the sensitivity to Eucalyptol and the levels of NO2, both known to be harmful pollutants^59,60^. PM 2.5 are formed to a large extent through gas phase reactions (unlike the PM 10 which are swept into the air like dust or sand). Typical constituents include ammonium, sulphate, nitrate as well as a variety of organic molecules all bundled up on the particle. Thus chemical composition of PM2.5 can be quite variable from place to place. This could also be attributed to a potential inaccurate representation of pollutants in the atmospheric model. The coarse spatial resolution of the model limits its ability to effectively resolve the gradients of pollutants, particularly in regions characterized by significant disparities between polluted and background areas. Perhaps lesser vulnerability to PM2.5 and NO2 species reflects the basic functionality of the trigeminal chemosensory system which is the detection of environmental hazards^61^. This could apply to relatively low concentrations of the given species, that are detectable but do not pose a threat to the functionality of olfactory receptors. Results from the clinical field point to possible bidirectional changes in trigeminal sensitivity as a differential effect of inflammatory reactions^58^. Hence, the future studies could look more closely on the nature of inflammatory reactions that may explain the already observed results in more detail. Critically, these results have to be considered in the context of the multifactorial model that we have tested. By no means our study allows to conclude beneficial effects of these chemical species for olfactory/trigeminal sensitivity. This would only hold true, if all other parameters in the model were constant and did not change. Air composition and the levels of chemical species constituting air pollution change dynamically. For instance, if the increase in PM2.5 is associated with an increase in PM10 ^62–64^, inflating the risk of multicollinearity. In the presented model PM10 has the strongest negative impact on olfactory/trigeminal sensitivity and the overall effect (combined with PM2.5) remains negative. In fact, a simplified statistical model including only three (PM2.5, O3 and NO2) out of six chemical species from the original model, consistently showed PM2.5 to be harmful to both olfactory/trigeminal (β = − 0.19, *p* < .001; Table S4A in Supplementary Materials) and olfactory (β = − 0.17, *p* < .001; Table S4B in Supplementary Materials; Table S3 for the simplified model fit coefficients). Therefore, when PM10, SO2 and CO were no longer included in the model, increased PM2.5 was related to lower chemosensory sensitivity. Hence, individual beta coefficients should always be interpreted in the context of the entire model. To reach ecologically valid conclusions, a wide variety of chemical species, resembling the atmospheric air, should be considered. Yet, this approach conveys a risk of multicollinearity, as many chemical species co-occur in the atmosphere and their levels are highly correlated. Therefore, future studies should involve some experimental efforts allowing control of chemical stimulation, and leading to causal, rather than correlational conclusions.

The effect sizes we have observed are small-to-moderate and should be interpreted in the context of the cumulative, rather than immediate, adverse impact of ambient air pollution on chemosensory health (Doty & Kamath^28^; Ekström et al.^30^; Olofsson et al.^65^). On the other hand, people who intoxicate their respiratory system for 10 years of smoking present similar olfactory performance with regard to naming odors, as people who do not share the smoking habit^66^. Therefore, interpretation of the adverse effects of toxic agents on the sense of smell should include a remark that the sense of smell may be relatively resilient to toxicity, probably due to its protected position inside the nose and its regenerative capacities^67^. More evidence on the protective role of nasal anatomy and neuroregeneration of the olfactory system is needed to propose a well-grounded perspective.

Age is considered a major demographic factor negatively affecting olfactory performance^68^. Olfactory performance decreases with age^65,69–72^ yet our data revealed a marginal increase in chemosensory thresholds with age in healthy men. However, beta coefficients for the relationship between chemosensory sensitivity and age are very small. They indicate that over the period of 80 years, men would gain 0.48 points and 0.11 points (for Eucalyptol and PEA respectively) in the 8-point threshold test, and this would be true only if no other factors undermining the sense of smell would occur. In practice, such change is unnoticeable for chemosensory perception and does not constitute an improvement significant in clinical terms^73^. The significance of the beta coefficient in the tested model is likely to arise from a large sample used in this study and should be interpreted with caution. Moreover, our total sample was relatively young. To draw more robust conclusions about chemosensory aging and pollution exposure, a study including older participants should be conducted.

By stepwise assessment of the statistical models, we demonstrated that previously reported variability in olfactory sensitivity across geographical locations^49^ can partially be attributed to the air composition in these regions. In other words, people exhibited different sensitivity to odors in different locations, partially because they were breathing air of different quality. This notion urges scientific efforts to examine olfactory sensitivity from a global perspective. Multicentre studies with such focus must put an emphasis on rigorous and highly standardized measurements of both olfactory sensitivity and air composition.

Conclusions from the current study offer new insights on chemosensory aging and the cumulative effects of air pollution on olfactory function in advanced age^28,30,70–72^. Our models point to medical conditions related to the sense of smell as a factor that accelerates olfactory aging and suppresses the gender-related differences noted among the healthy population^74–76^. Finally, the study corroborates seasonal fluctuation in chemosensory sensitivity by showing that scores obtained in the Autumn and Winter were relatively lower than those obtained in Spring and Summer. These effects can be attributed to more frequent upper respiratory tract infections that are known to hinder olfaction and are subject to seasonal fluctuation^77^. Another explanation of seasonal changes in chemosensory sensitivity may refer to the seasonal fluctuations in the levels of particulate matter (PM). Although the seasonal peak of pollutants depends on the location, in urban environment the levels of PM2.5 and PM10 are known to increase in the Autumn and Winter, likely resulting from heating systems or traffic emissions^78^. On the other side, PM10 particles are mostly primary particles such as soil dust, sand, soot, or sea salt, which are emitted directly into the atmosphere, usually by the wind. Therefore, in background areas, their peaks may be observed in the summer^79^. In the current study, we tested chemosensory sensitivity in urban settings and slightly lower scores were obtained in the Autumn and Winter, likely due to the increased pollution levels. It is recommended that both season (or even more precise characteristics such as humidity and temperature) and location should be accounted for in future studies concerning chemosensory sensitivity and air pollution. More clear understanding of the interaction between these two factors is in demand.

The present study has certain limitations that require reflection. Participants included in this study were mostly urban-dwellers. Although previous studies confirmed that urban populations are the most exposed to environmental pollution and suffer more often from olfactory deficits^7,17,18,29,80,81^ it would be recommendable to collect more data among populations breathing air of better quality to improve the predictions. It is specifically important given the fact, that clean air of good quality becomes a luxury enjoyed by the minority of the human population. Furthermore, the study does not capture the longterm pollution exposure, but focuses on the outdoor pollution on the day of the test. The study investigated the correlations between the levels of specific chemical species associated with air pollution and chemosensory sensitivity. However, the study design does not permit making causal statements. Although we were able to construct quite complex models and tested them systematically against each other, they are still somehow simplification of the complex nature of the relationship between atmospheric air human chemosensory sensitivity. In particular, we treated the data seasonally and the current study does not capture the day-to-day variability in the chemical composition of the air. Thus, we demonstrated the influence of general trends in the annual variation of the pollutant species. The present study does not capture local or occasional effects (e.g. barbecue, construction sites, heating season, etc.). There seem to be at least two ways to capture the complexity of air quality even better. One approach to address this limitation would involve incorporating a broader range of chemical species into the statistical model. However, it is important to recognize the potential for inconclusive results numerous interactions and autocorrelations present within the air composition which could undermine the validity of our results, as previously noted. Thus, particular attention should be given to (1) the pollutants investigated (2) the model used for estimating their relationship. Another way would be to use personal air sensors on each individual subject to monitor the exact air composition they are breathing in or measure and standardize the air composition in the testing rooms. These would be technically challenging, yet fascinating studies.

## Conclusions

Our investigation extends and deepens our understanding on the harmful effects of air pollution on olfactory sensitivity and extends it by demonstrating that the trigeminal/olfactory sensitivity as well suffers from toxic air, but in a slightly different manner. Furthermore, the presented models promote a more complex perspective on the relationship between air composition and chemosensory sensitivity. Conclusions point to the need to investigate the problem of air pollution and chemosensory health from the global perspective, as air quality partly accounts for the initial differences in chemosensory perception in different regions of the world. With this investigation, we hope to create awareness of the challenges for research on air pollution and olfactory sensitivity and we urge interdisciplinary research efforts to monitor the air composition even more precisely.

### Ethical considerations

Chemosensory sensitivity testing was performed in accordance with the Declaration of Helsinki on Biomedical Studies Involving Human Subjects. Informed written consent was obtained from all participants. Empirical data from individual human participants were then matched with the air quality records according to the geographical coordinates of each local study site and the date of testing. The entire study design and consent approach were approved by the Ethics Review Board at the University of Wroclaw (3/2021), Ethics Review Board at University Clinic of the TU Dresden (EK354092017) and the local IRBs when required.

### Participants

The total sample comprised 711 participants (480 females) aged between 17 and 61 years. Compared to the 802 participants described earlier^49^ we excluded 91 participants due to the missing data for self-assessed olfactory function (*n* = 90) or psychophysical ratings (*n* = 1). Descriptive statistics for age and the proportion of females in each country subscale are presented in Table 2.

**Table 2:** Proportion of gender and descriptive statistics for age in the total sample in each of the study locations.

Country	N	% Females	Age				
			Mean	SD	Median	Min	Max
Australia	74	60.81%	19.9	4.15	18	17	40
China	80	52.50%	31.6	11.1	26	19	56
Germany	72	62.50%	28.6	9.45	24	19	56
India	32	53.13%	40.9	13.6	43.5	19	55
Iran	100	74.00%	32.3	9.68	31	18	55
Japan	82	48.78%	29.1	9.82	26.5	19	55
Mexico	80	50.00%	19.9	2.73	19	18	34
Poland	80	50.00%	27.1	7.47	25	18	53
Turkey	45	51.11%	29.5	4.63	29	23	39
USA	66	78.79%	36.7	12.5	33	21	61
Total sample	711	59.90%	28.8	10.6	25	17	61

One-hundred eighty-nine participants reported medical conditions that could potentially influence their olfactory performance (e.g. allergic rhinitis, chronic rhinosinusitis, hyperthyroidism, hypertension) and this information was included in the models predicting olfactory/trigeminal and olfactory sensitivity. All participants were recruited among urban dwellers, usually inhabiting cities where partnering laboratories were located. Approximately half of the sample was recruited from the general population and the remaining participants were students. They were recruited via local advertisements or constituted convenience samples.

## Materials and methods

The aim of the study was to match the empirical data on chemosensory sensitivity (published earlier in: Oleszkiewicz et al.^49^) with the secondary data on air pollution indices from the testing sites.

### Chemosensory sensitivity testing procedure

Data were collected in ten different study locations according to the following guidelines. Each subject was tested during an individual session in well-ventilated, air-conditioned rooms. All participants read introductory instructions to the study and provided basic demographic information. Further, they were given a standard medical interview (Welge-Luessen et al. 2013) to identify participants with a medical history that could potentially alter olfactory/trigeminal sensitivity. Next, chemosensory tests were performed in a randomized order with 15 min break in between^82^. One test utilized Eucalyptol (eucalyptus odor) which is known to activate both olfactory and trigeminal systems^83^. We used it to examine olfactory/trigeminal sensitivity. The other test contained phenylethyl alcohol (PEA; rose odor) known to be a relatively selective olfactory stimulant (with little trigeminal activity; Doty et al.^84^), and here we used it for testing olfactory sensitivity. At the end of the session, participants provided their psychophysical ratings of Eucalyptol and PEA and rated their olfactory sensitivity on the testing day in relation to their usual olfactory performance. The order of these two tasks was randomized.

### Testing materials

All contributing laboratories were provided with custom-made threshold tests manufactured in the Smell and Taste Center, TU Dresden. Odors were presented with felt-tip pens (Sniffin’ Sticks) of approximately 14 cm in length and an inner diameter of 1.3 cm filled with 4 ml of liquid odorant. To present an odor, the experimenter removes the cap from the pen for approximately 3s, brings the pen’s tip in front of the subject’s nose, and carefully moves it from left to right nostril and backward. The threshold scores were obtained using a three alternative forced choice paradigm. Participants were repeatedly presented with triplets of pens (target pen with odor + 2 odorless pens). Starting with the lowest odor concentration, a staircase paradigm was used where two subsequent correct indications of the odorous pen or one incorrect answer marked a so-called turning point and resulted in a decrease or increase, respectively, of concentration in the next triplet. Triplets were presented at 20s intervals^85^. The threshold score is the mean of the last four turning points in the staircase, with the final score ranging between 1 and 8 points. In our study, the highest concentration for PEA was a 0.25% odor solution whereas the highest concentration for Eucalyptol was 0.03125%. Eight concentrations were created by stepwise diluting previous ones by 1:3.

For the psychophysical odor ratings of pleasantness, intensity and familiarity we used 7-point Likert-type scales ranging from 1-*not at all* to 7-*very* (e.g. not intense at all – very intense). We also included the measurement of self-rated olfactory function with the 7-point Likert-type scale ranging from 1-*very much worse* to 7-*very much better.* The reference category was the usual own olfactory performance. The order of psychophysical ratings and self-assessment was randomized.

### Air pollution data retraction

The pollution data were obtained from a numerical simulation with the EMAC (ECHAM5/MESSy for Atmospheric Chemistry) model^86^. The EMAC simulation used is described in detail in^87^ and here only the most important details are presented. The EMAC simulation has a horizontal resolution of T63 grid, corresponding to a quadratic Gaussian grid of approximately ~ 1.8° × 1.8° degrees in latitude and longitude, with 47 hybrid vertical levels up to 0.01 hPa. The model simulation, nudged towards the ERA-5 meteorological re-analyses, was performed from January 2017 to December 2020, so to encompass all the period of the chemosensory sensitivity test. The monthly varying anthropogenic emission inventory CAMS-GLOB-ANTv4.2^88^ were used for the primary emitted species. Biomass burning emissions were obtained from the Global Fire Assimilation System (GFAS) inventory^89^. Over the past decade, EMAC model simulations of aerosols and trace gases have been extensively assessed against ground measurements and satellite retrievals^90–92^. The daily averages of the pollutants were extracted for the corresponding location (with bilinear interpolation from the original model grid) and the day of the chemosensory sensitivity test. Nevertheless, it is important to emphasize the significant uncertainties associated with the coarse resolution of global models, leading to high uncertainties in estimating the mixing ratios (or concentrations) of aerosols and trace gases. This is especially true for substances such as NO2, which exhibit substantial sub-grid variability^93^.

Descriptive statistics for the pollution levels across the locations and average olfactory/trigeminal and olfactory threshold scores are given in Table S1.

### Statistical analyses

Analyses were conducted using R (R Team^94^) and IBM SPSS v27 (IBM, Chicago, SPSS Inc.). The exact date and time of the measurements were too precise to allow generalization of the conclusions, therefore it was converted into *Season* representing the meteorological season of the year, accounting for the differences between the northern and southern hemispheres. The final threshold score was the mean of the last four reversals in the staircase measurement. Thus, an individual subject could score one of the 29 possible scores ranging from 1 to 8 with an interval of every 0.25 points. We transformed these raw scores to a ratio scale of 0–28 points with the formula $\:(X-1)\cdot\:4$. We regressed individual and geographical factors on olfactory and olfactory/trigeminal thresholds by constructing the base model and supplementing it stepwise with self-rated olfactory function, location, and air pollution. We chose the stepwise regression model due to the numerous predictors. The stepwise approach allows entering only independent predictors that improved the model and omitting irrelevant predictors, at the same time correcting the estimates for multiple predictors.

We constructed two baseline models – one for olfactory/trigeminal threshold (Eucalyptol) and one for the olfactory threshold (PEA). The baseline models included individual factors: Age, Gender (reference: female); Smell-related medical conditions (reference: no), Pleasantness, Intensity, and Familiarity ratings of the suprathreshold concentration of the threshold odor. Interaction terms for the two binominal predictors, i.e. Gender and Medical conditions were included in the baseline models. Further, we compared each of the baseline models with seven alternative models including (or not): Self-assessed olfactory function, Season (reference: Spring) and Location (reference: Poland), Air pollution (indices were six chemical species: PM2.5, PM10, O3, NO2, SO2, CO). We estimated predictors using the leave-one-out cross-validation method by fitting the model sequentially without each observation and comparing its fitness. The significance of the change in the model fit was assessed with Akaike’s Information Criterion (AIC). This makes the fit quality measures robust to model over-fitting. MAD (Mean Absolute Deviation) and Pearson’s linear correlation coefficient were used to assess model quality. Holm-Bonferroni correction for multiple comparisons was used to assess the significance of variables to avoid an excess of false positives with potentially hundreds of regression model parameters.

## Electronic supplementary material

Below is the link to the electronic supplementary material.

Supplementary Material 1

## Data Availability

Raw data for this project are publicly available at https://osf.io/m95z4/.
